# Robust deep learning model for prognostic stratification of pancreatic ductal adenocarcinoma patients

**DOI:** 10.1016/j.isci.2021.103415

**Published:** 2021-11-10

**Authors:** Jie Ju, Leonoor V. Wismans, Dana A.M. Mustafa, Marcel J.T. Reinders, Casper H.J. van Eijck, Andrew P. Stubbs, Yunlei Li

**Affiliations:** 1 Department of Pathology & Clinical Bioinformatics, Erasmus MC Cancer Institute, University Medical Center Rotterdam, Rotterdam, the Netherlands; 2 Department of Surgery, Erasmus MC Cancer Institute, University Medical Center Rotterdam, Rotterdam, the Netherlands; 3 The Delft Bioinformatics Lab, Delft University of Technology, Rotterdam, the Netherlands

**Keywords:** Biocomputational method, Cancer systems biology, Cancer

## Abstract

A major challenge for treating patients with pancreatic ductal adenocarcinoma (PDAC) is the unpredictability of their prognoses due to high heterogeneity. We present Multi-Omics DEep Learning for Prognosis-correlated subtyping (MODEL-P) to identify PDAC subtypes and to predict prognoses of new patients. MODEL-P was trained on autoencoder integrated multi-omics of 146 patients with PDAC together with their survival outcome. Using MODEL-P, we identified two PDAC subtypes with distinct survival outcomes (median survival 10.1 and 22.7 months, respectively, log rank p = 1 × 10^−6^), which correspond to DNA damage repair and immune response. We rigorously validated MODEL-P by stratifying patients in five independent datasets into these two survival groups and achieved significant survival difference, which is superior to current practice and other subtyping schemas. We believe the subtype-specific signatures would facilitate PDAC pathogenesis discovery, and MODEL-P can provide clinicians the prognoses information in the treatment decision-making to better gauge the benefits versus the risks.

## Highlights

• We developed DL-based MODEL-P to identify prognosis-correlated PDAC subtypes
• The identified subtypes related to DNA damage repair and immune response processes
• MODEL-P stratified patients from independent datasets into distinct survival groups
• MODEL-P could be used in clinics to aid treatment decision-making

## Graphical abstract

**Figure:**
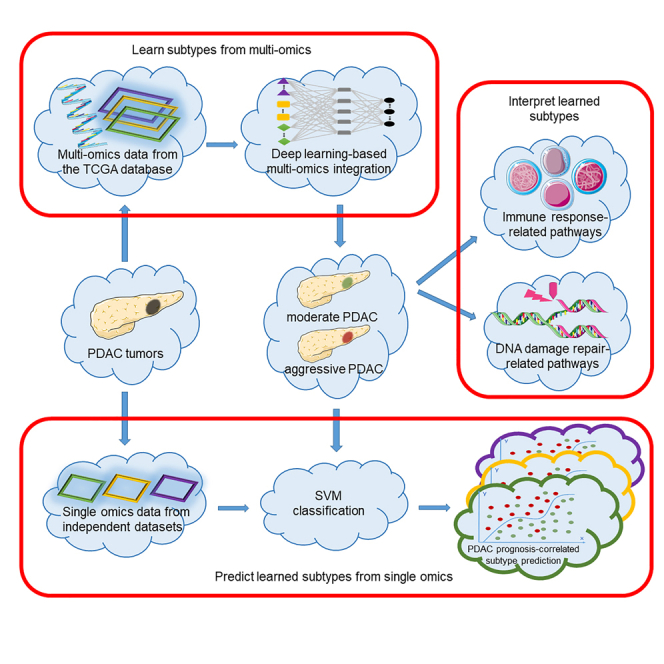

## Introduction

Pancreatic cancer is the third leading cancer-related cause of death worldwide, with a 5-year survival rate of only 9% (Siegel et al., 2020). Pancreatic ductal adenocarcinoma (PDAC) accounts for 85% of all pancreatic cancers and is the most aggressive subtype. One of the major clinical challenges is the heterogeneity of PDAC. Patients have diverse oncogenesis and distinct survival outcomes leading to inaccurate diagnosis and improper treatment (Ryan et al., 2014). Current medical and molecular tests only provide limited information on tumor aggressiveness and patient prognosis to make a personalized treatment plan. Therefore, the prognosis after surgery remains unpredictable (Guillén-Ponce et al., 2017). For example, computed tomography (CT) scan, one of the most commonly used methods to help tumor diagnosis, only provides information on tumor stage, based on which the clinicians infer the tumor or metastatic lesions and consequently assess the possibility for surgery. Another widely used tumor marker for pancreatic cancer prognosis prediction is carbohydrate antigen (CA) 19-9, which is tested in blood. Although the changes of its secretion indicate the progress of pancreatic cancer and enable monitoring of treatment response, CA 19-9 is not recommended to be used solely to determine operability or predict recurrence or treatment response owing to the high false-positive and false-negative results (Guillén-Ponce et al., 2017; Locker et al., 2006).

To improve patient care and provide more effective therapeutic plans, bioinformatics approaches have been developed to define PDAC subtypes from molecular perspectives enabling personalized diagnosis and treatment (Aguirre, 2018; Bailey et al., 2016; Collisson et al., 2011; Golan and Javle, 2017; Grant et al., 2016; Moffitt et al., 2015; Nicolle et al., 2017; Sinkala et al., 2020). However, unsupervised clustering of patients with PDAC performed on different types of omics separately (i.e., single omics), such as genomics, transcriptomics, and proteomics, may result in subtypes with highly inconsistent patient classification (Sinkala et al., 2020; Wang et al., 2014). This is because each type of omics data contains unique information of PDAC and links to different oncogenesis and tumor development mechanisms. To take it one step further, we need tools that identify unified subtypes from multi-omics such that molecules that are associated with similar biological process are aggregated and subsequent single omics-based analysis is strengthened (Canzler et al., 2020; de Anda-Jáuregui and Hernández-Lemus, 2020; Koh et al., 2019; Nicora et al., 2020; Ulfenborg, 2019). iCluster robustly identified subtypes for breast cancer and lung cancer by integrating gene expression and copy number variation data (Shen et al., 2009) and has shown that the multi-omics-based subtypes are more informative than those resulting from single omics. Similarity Networks Fusion (SNF), which builds a similarity matrix of samples within and across different types of omics, also has been applied to pancreatic cancer to identify molecular subtypes based on proteins, mRNAs, DNA methylation, and microRNA profiles (Sinkala et al., 2020). Although the unsupervised subtyping revealed molecular diversity of patients with PDAC, the patients within each subtype still had a broad spectrum of survival outcomes, such that the prognosis differences among subtypes are not significant (Aguirre, 2018; Sinkala et al., 2020). These data suggest that within each subtype patients may still have different levels of tumor aggressiveness. Therefore, there is an urgent need for more refined prognosis prediction to facilitate precise decision-making on treatment plans, deliver more efficient therapy, and diminish the negative effects from unnecessary intervention.

In this study we proposed a deep learning-based framework—Multi-Omics DEep Learning for Prognosis correlated subtyping (MODEL-P)—to create prognostic relevant PDAC subtypes that can then be used to stratify patients with PDAC into different survival risk groups. A similar multi-omics integration framework was proposed to identify liver cancer patient subtypes with distinct prognosis (Chaudhary et al., 2018). In addition, we identified the subtype signatures for future patient prognosis prediction and for subtype-specific biological processes exploration. Of importance, we demonstrated that the utility of MODEL-P to unify PDAC subtyping improved prognosis prediction.

## Results

Before the development of MODEL-P, we collected mRNA sequencing (mRNA-seq), microRNA sequencing, DNA methylation array, and clinical information (Table 1) of 146 PDAC surgically resected primary infiltrating (non-metastatic) patients from The Cancer Genome Atlas (TCGA) PAAD cohort (Raphael et al., 2017). This multi-omics dataset served as our training set, and we obtained five datasets as external single omics test sets. Three of these came from the International Cancer Genome Consortium (ICGC) Australian cohort (2010) (one mRNA-seq, one mRNA microarray, and one DNA methylation dataset). Two of these test sets came from the Gene Expression Omnibus (GEO) database (Yang et al., 2016) (one mRNA array and one microRNA dataset) (see key resources table).

**Table 1:** Association between the clinical factors and identified subtypes

Clinical factor (#patients)		Values	“Moderate” subtype	“Aggressive” subtype	*p* values
Follow-up time after diagnosis (months) n = 146		11.5 ± 13.7	7.9 ± 5.7	13.0 ± 15.7	0.42
Resected tumor size (cm) n = 134		3.8 ± 1.4	3.5 ± 1.1	4.3 ± 1.7	0.011
Age (year) n = 146		65.2 ± 10.9	64.2 ± 10.9	67.5 ± 10.6	0.11
Survival status n = 146	Alive	91 (62.3%)	71	20	0.015
	Deceased	55 (37.7%)	32	23	
2-year overall survival n = 146	>2 year	15 (10.3%)	15	0	0.0057
	≤2 year	131 (89.7%)	88	43	
Gender n = 146	Female	68 (46.6%)	55	13	0.011
	Male	78 (53.4%)	48	30	
Tobacco usage n = 118	Current non-smoker	101 (85.6%)	71	30	0.17
	Current smoker	17 (14.4%)	9	8	
Alcohol usage n = 134	Never	50 (37.3%)	39	11	0.12
	Yes	84 (62.7%)	54	30	
History of diabetes n = 121	No	88 (72.7%)	62	26	0.2
	Yes	33 (27.3%)	19	14	
History of chronic pancreatitis n = 116	No	105 (90.5%)	70	34	1
	Yes	11 (9.5%)	7	4	
T n = 145	T1	4 (2.8%)	3	1	1
	T2	15 (10.3%)	11	4	
	T3	123 (84.8%)	86	37	
	T4	3 (2.1%)	2	1	
N n = 145	N0	37 (25.3%)	24	13	0.36
	N1	108 (74.0%)	79	29	
M n = 72	M0	69 (47.3%)	47	22	1
	M1	3 (2.0%)	2	1	
Residual tumor n = 136	R0	83 (61.0%)	61	22	0.54
	R1	47 (34.6%)	29	18	
	R2	6 (4.4%)	4	1	
AJCC tumor stage n = 145	Stage I	12 (8.3%)	7	5	0.8
	Stage II	127 (87.5%)	91	36	
	Stage III	3 (2.1%)	2	1	
	Stage IV	3 (2.1%)	2	1	
Neoplasm histologic grade n = 146	G1	21 (14.4%)	18	3	0.11
	G2	83 (56.8%)	60	23	
	G3	41 (28.1%)	24	17	
	G4	1 (0.7%)	1	0	

The mean +/− standard deviation values of numerical variables and the number of patients falling into each of the categorical variables were given. The associations between the clinical factors and the MODEL-P subtypes were presented by the *p* values of Mann-Whitney U test for numerical variables (follow-up time after diagnosis, resected tumor size, age) and Fisher's exact test for categorical variables (the other clinical factors).

**Figure 1:**
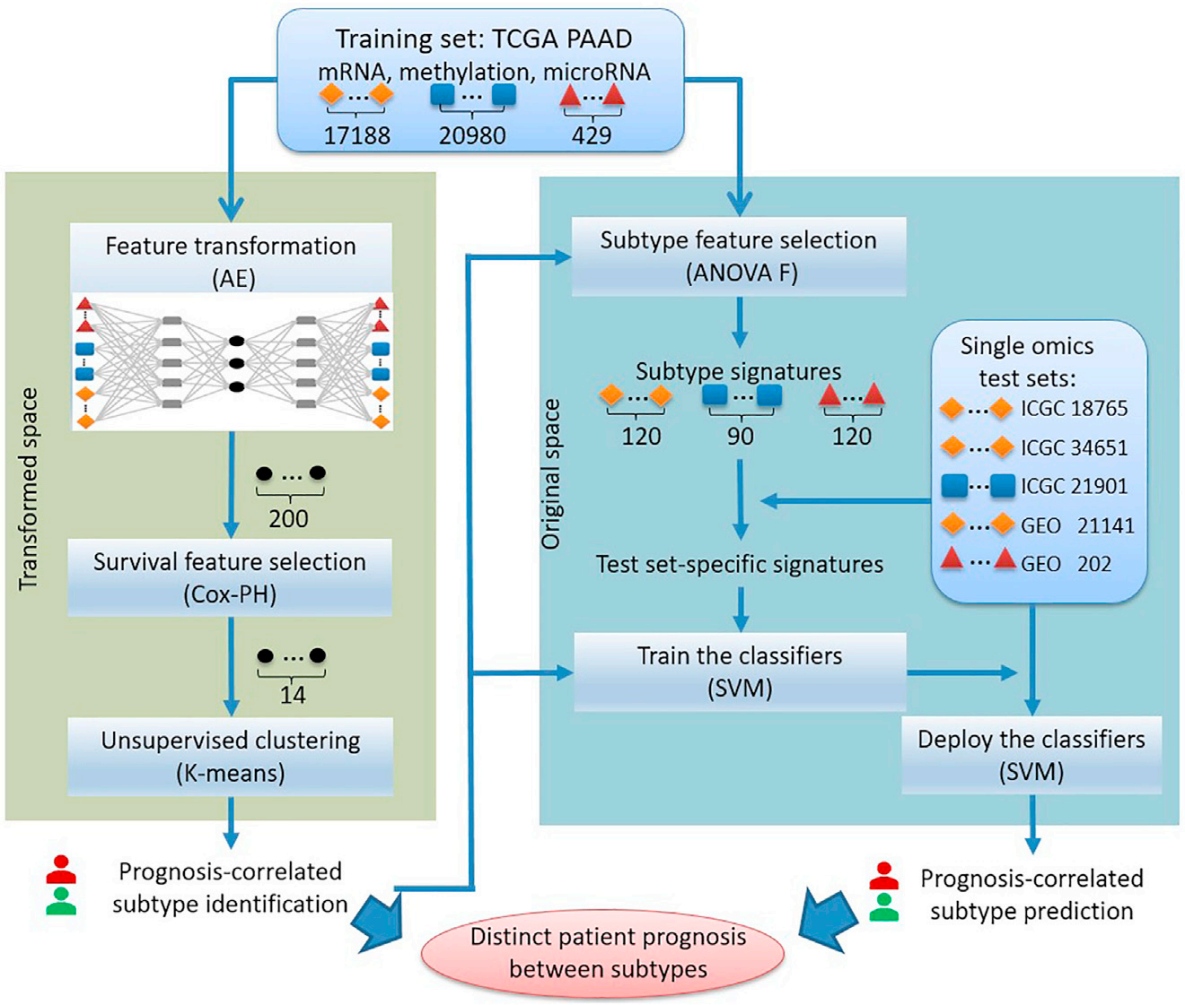
Study design of MODEL-P First, the multi-omics features in the training set were integrated by autoencoder (AE), after which the transformed features were selected with regard to survival outcomes for clustering to identify the prognosis-correlated subtypes. Second, the features in the original space that differ between the prognosis-correlated subtypes were selected as subtype signatures. Afterward, we selected those subtype signatures that were present in the test sets as the test set-specific signatures. The classifiers were trained on the TCGA training set using these signatures and the predictions were made on the corresponding test sets. The numbers of features are given for each data type. AE, autoencoder; Cox-PH, Cox Proportional-Hazards model; SVM, support vector machine.

Then MODEL-P (Figure 1) was developed to first identify PDAC subtypes that correlate with patients' prognosis. Based on the identified subtypes and associated subtype signatures, our second aim was to predict patients' prognosis and stratify them into distinct survival risk groups. For the first aim, prognosis-correlated subtypes were defined based on the integrated TCGA multi-omics training set. More specifically, an autoencoder (AE) model was constructed to transform and integrate multi-omics features. This was followed by feature selection in the transformed feature space with regard to survival outcomes. Afterward, *K*-means clustering was performed to identify prognosis-correlated PDAC subtypes. For the second aim, each patient from the single omics test sets was classified into one of the identified prognosis groups. To do so, we first deduced the subtype signatures in the original space for each data type separately and used those overlapping omics between the subtype signatures and the test set at hand as the test set-specific predictors. Afterward, Support Vector Machine (SVM) (Cortes and Vapnik, 1995) classifiers were constructed on the TCGA training set using the prognosis-correlated class labels identified above and the test set-specific predictors. The trained classifiers were deployed on the patients from the five external test sets to classify them into distinct prognosis subgroups.

### PDAC prognosis subtype identification and prediction

**Figure 2:**
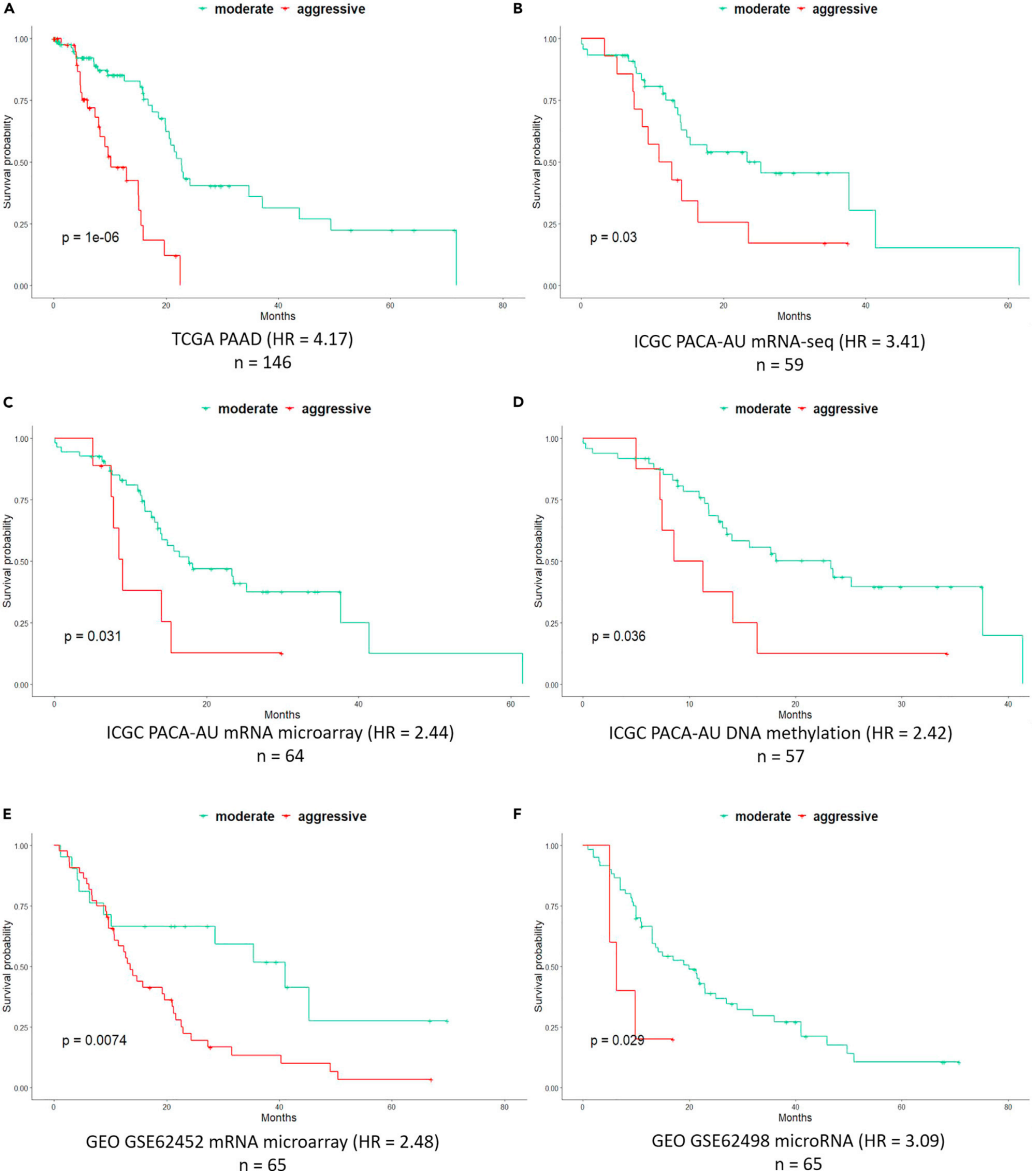
Results of PDAC prognosis subtype identification and prediction (A) Kaplan-Meier plot of two prognosis-correlated subtypes identified in the TCGA PAAD cohort, with a log rank p value of 1 × 10^−6^ and the hazard ratio of 4.17. (B–F) Kaplan-Meier plots of the prognosis-correlated subgroups predicted on five single omics test sets: (B). ICGC PACA-AU mRNA-seq, (C). ICGC PACA-AU mRNA microarray, (D) ICGC PACA-AU DNA methylation, (E). GEO GSE62452 mRNA microarray, (F) GEO GSE62498 microRNA. The log rank p values of the datasets are given in each individual plot, together with the name of the datasets, the sample sizes, and the hazard ratios below the plots.

In the subtype identification phase carried out on the TCGA training set, the AE model generated 200 integrated features from the multi-omics data. After features selection, the 14 prognosis-correlated features were used to identify prognosis-correlated subtypes by performing k-means clustering. The number of optimized clusters was determined by the results of the silhouette width (Rousseeuw, 1987) and Calinski-Harabasz criterion (Calinski and Harabasz, 1974) metrics, which consistently suggested that there were two clusters present in the integrated multi-omics training set (Figure S1). We refer to these two clusters as “aggressive” and “moderate”. They have significantly different overall survival (OS) in the TCGA training set with a log rank *p* value of 1 × 10^−6^ (Figure 2A). The “moderate” subtype consists of 103 patients with a median survival time of 22.7 months, and the “aggressive” subtype consists of 43 patients with a median survival time of 10.1 months. Moreover, the hazard ratio (HR) between the “aggressive” subtype and the “moderate” subtype was 4.17 (*p* value = 4.89 × 10^−6^).

In the prognosis prediction phase, predictors of the MODEL-P subtypes were identified for each single omics data type and tested on the single omics external datasets to stratify patients into subgroups with distinct survival outcomes. As a result, 120 mRNA features, 120 microRNA features, and 90 DNA methylation features were selected as the subtype signatures (Data S1). The prediction results are shown in Figures 2B–2F and Table S1. Patients were classified into two prognosis-correlated subtypes with log rank *p* values of 0.030, 0.031, 0.036, 0.007, and 0.029 for ICGC mRNA-seq, ICGC mRNA microarray, ICGC DNA methylation, GEO: GSE62452 mRNA microarray, and GEO: GSE62498 microRNA dataset, respectively.

### Single omics contribution

**Figure 3:**
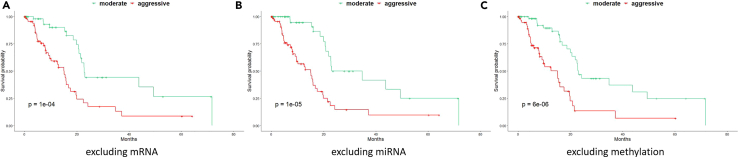
Contributions of mRNA, microRNA, and methylation omics to subtype identification in TCGA PAAD cohort (A–C) In each Kaplan-Meier plot, the two subtypes were identified excluding (A) mRNA (log rank *p* value = 1 × 10^−4^), (B) microRNA (log rank *p* value = 1 × 10^−5^), or (C) methylation (log rank *p* value = 6 × 10^−6^). Note that a larger p value here indicates that leaving out that data type reduces the prognostic performance the most, i.e. the results need to be compared with Figure 2A.

We investigated the contribution of each different type of omics in the PDAC subtype identification by removing one type of single omics at a time from the input before data integration and compared the prognostic power of the MODEL-P clusters with and without this type of omics. We demonstrated that the identified subtypes became less distinct after removing mRNA sequencing (log rank *p* value = 1 × 10^−4^), followed by microRNA data (log rank *p* value = 1 × 10^−5^), and then DNA methylation (log rank *p* value = 6 × 10^−6^). This indicates that the mRNA profiling is more informative than the other two omics features to define the PDAC prognosis subtypes (Figure 3). Subtypes are best identified when integrating the three types of omics data as it leads to the largest survival difference between the stratified groups (log rank *p* = 1 × 10^−6^, Figure 2A).

### Unsupervised subtype identification

To illustrate the value of supervised feature selection for subtyping, we also conducted an unsupervised subtyping. That is, after AE feature integration, the 200 transformed features were used to perform a *K*-means clustering directly without any prognosis-dependent feature selection. As a result, the unsupervised subtyping identified two subtypes with a log rank *p* value of 0.005 for the TCGA training set, which is less significant than MODEL-P using the supervised feature selection (log rank *p* value = 1 × 10^−6^). As this result is biased for the supervised approach, we next evaluated the prediction performance on the external datasets when the single omics features are chosen on the basis of the unsupervised subtypes. None of the predictors were able to stratify patients on prognosis (log rank *p* values > 0.05, Table S1). This shows that the supervised feature selection is an important contribution within MODEL-P as it makes the clusters prognosis aware.

### AE versus other feature extraction methods

To compare the feature selection by our proposed AE approach with other popular feature extraction methods, we replaced the AE by either principal component analysis (PCA) or non-negative matrix factorization (NMF) (Jolliffe and Cadima, 2016; Lee and Seung, 1999) in our framework and repeated the identification of PDAC subtypes. The optimal parameters and selected features were tuned in the same way as the AE model construction. Both PCA (with 100 principal components and 7 prognosis-correlated features) as well as NMF (with 50 non-negative elements of factorized matrix and 6 prognosis-correlated features) generated subtypes with significant log rank *p* values to stratify PDAC prognosis in the TCGA cohort (0.012 and 0.003, respectively, Figure S2), but these results are worse when compared with our AE feature selection approach (log rank *p* = 1 × 10^−6^). This suggests that one can benefit from (a learned) non-linear feature selection as was done by the AE.

### Impact of clinical factors

To further understand and improve MODEL-P for multi-omics-based prognosis-correlated subtype identification, we also considered the role of clinical factors, such as patient and tumor characteristics. Three analyses were performed on the TCGA training set for 146 non-metastatic patients who underwent surgical resection for PDAC. We included clinical information available in the TCGA training set that could potentially influence the prognosis: current tobacco usage, age, gender, alcohol usage, diabetes diagnosis, chronic pancreatitis, TNM classification of malignant tumors (TNM staging, including T, N, and M classification individually), neoplasm histologic grade, and residual tumor.

First, we assessed the association between the clinical factors above and the identified MODEL-P subtypes using Fisher's exact test with fisher.test function in R (Table 1). This analysis showed that only “gender” was associated with the subtypes identified in this study (*p* = 0.011). Men were more often associated with the “aggressive” subtype than women, which is also supported in the literature: men have significantly higher hazard ratios in both localized (T-stages 1/2) and extended (T-stages 3/4) PDAC (Gleason et al., 2013; Rawla et al., 2019).

Second, we used the clinical factors one at a time in a univariate Cox-PH model to assess their correlations with the OS. The univariate model showed that patients with microscopic residual tumor (R1) after pancreatic surgery had a significantly worse prognosis than patients with no residual tumor (R0, HR = 2.37, *p* value = 0.004). For macroscopic residual tumor (R2) factor, no statistically significant hazard ratio was observed owing to the very small sample size of five (Figure S3).

Third, we combined one of the factors each time together with the MODEL-P-identified subtypes in a multivariate Cox-PH model to assess their combined prognostic ability. The multivariate Cox-PH model showed that, within each subtype, patients with microscopic residual tumor (R1) had worse survival outcomes than those with no residual tumor (R0, HR = 2.63, *p* value = 0.002) (Figure S4). Postoperative residual tumor status, as the only significant factor in this multivariate analysis, has been frequently described in the literature with R1 status being associated with a worse survival than patients with R0 status (Tian et al., 2019). None of the preoperative clinical risk factors had added value on top of MODEL-P subtypes for patient prognosis prediction, also suggesting that all information in preoperative clinical factors had been covered by molecular profiling. Moreover, the MODEL-P subtypes remained to be a strong predictor of prognosis (HR = 4.27, p value = 1.4 × 10^−5^) independent of the postoperative residual tumor status.

### Pathway analysis

To understand the biological processes and pathways related to our identified PDAC subtypes, a gene set enrichment analysis (GSEA) (Subramanian et al., 2005) was performed based on the mRNA expressions. Within GSEA, the differentially expressed genes (DEGs) between the “aggressive” and “moderate” subtypes were identified and used to query against the Molecular Signatures Database representing Kyoto Encyclopedia of Genes and Genomes (KEGG) (Kanehisa et al., 2021) pathways and Gene Oncology (GO) (Ashburner et al., 2000) terms.

**Figure 4:**
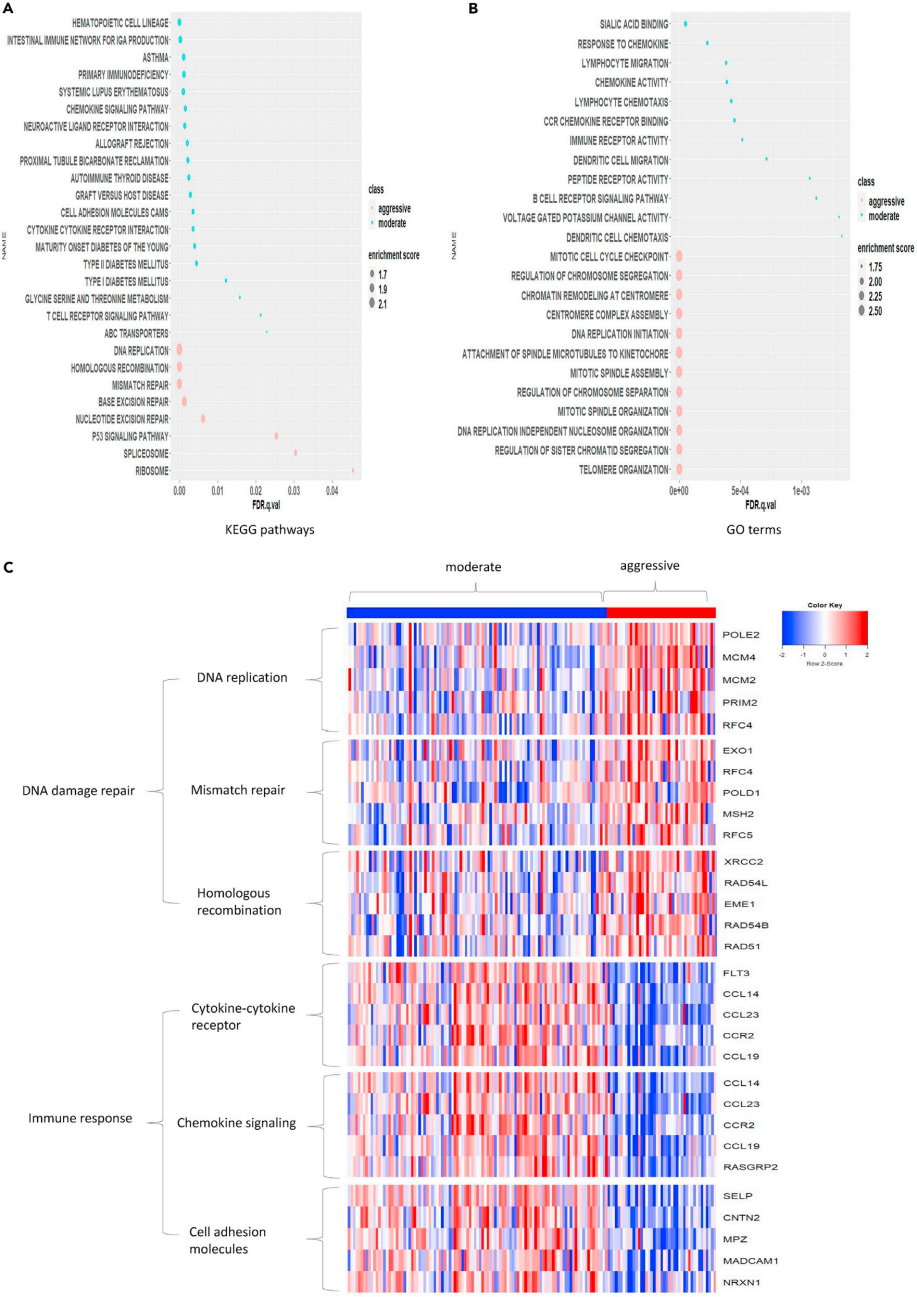
KEGG pathways and biological processes enriched in PDAC “aggressive” and “moderate” subtypes identified from mRNA expressions on the TCGA training set (A) The KEGG pathways. (B) The top 12 GO terms. For A and B, the size of each circle represents the absolute value of the normalized enrichment scores and the color represents the subtype enriched in PDAC “aggressive” and “moderate” subtypes. (C) Heatmap of the KEGG pathways corresponding to DNA damage repair and immune response in “aggressive” and “moderate” subtypes, respectively. The top five ranked genes were given in the panel.

The resulting pathways/GO terms were significantly enriched in the two subtypes but regulated to different extents. Twenty-seven KEGG pathways were identified from the mRNA dataset (Figure 4A) with |normalized enrichment score (NES)|> 1.5 and FDR *p* value < 0.05. The top 12 reported biological processes GO terms identified from mRNA dataset are shown in Figure 4B. The significant KEGG pathways and GO terms that were found more activated in the “aggressive” subtype fall into the category of DNA repair mechanism (Figure 4A) and DNA replication (Figure 4B). DNA damage repair (DDR) pathways include overlapping pathways of DNA replication, homologous recombination, and mismatch repair. We also show the top five most differentially expressed genes between the two subtypes in each of the DDR-related pathways (Figure 4C). DNA replication GO terms include DNA replication initiation and centromere complex assembly. Thus, both the mRNA-based KEGG pathways and GO terms results demonstrate that the “aggressive” subtype tumor progression involves relatively up-regulated DDR mechanisms in combination with increased mitosis, which would promote tumor growth. In addition, the identification of upregulated P53 signaling pathway in the “aggressive” subtype, which is activated by the stress signals like DNA damage, oxidative stress, and activated oncogenes (Liu and Xu, 2011), supports the role of DDR pathways in this subtype.

The “moderate” subtype was mainly characterized by relatively up-regulated pathways related to immune response, including chemokine signaling (Lee and Rhee, 2017), cell adhesion molecules (CAMs) pathway (Farahani et al., 2014), cytokine-cytokine receptor interaction pathway (Lee and Rhee, 2017) (Figure 4A) and response to chemokines, dendritic cell migration, and B cell biological processes (Figure 4B). Similarly, we indicated the top five most differentially expressed genes in each of the immune response-related pathways in Figure 4C.

### Single-nucleotide variants associated with the subtypes

To further understand the genetic basis of the prognosis-correlated subtypes, we conducted two analyses on the single nucleotide variants (SNVs) data from the TCGA PAAD cohort containing 140 samples.

First, we analyzed the association between SNVs and the identified MODEL-P subtypes on the TCGA dataset by Fisher's exact tests. The SNV data were available for 140 PDAC samples and 20,163 SNVs in total, which consist of 9,966 genes with five types of mutation (“missense”, “nonsense”, “nonstop”, “silent”, “splice site”) if available. Since the occurrence of the SNVs is extremely sparse (i.e., 99.8% of the SNVs occur in at most one sample), we excluded all these SNVs (with occurrence ≤1 sample) before testing to increase the statistical power. This left us with 41 SNVs in 30 genes that were subjected to a Fisher's exact test with an FDR cutoff of 0.05.

Only one significant association between the identified subtypes and the SNVs was found, being a KRAS missense mutation (rs121913529, p value = 4.25 × 10^−5^, FDR = 0.002), which also has been reported in the TCGA study (Raphael et al., 2017). Specifically, for the KRAS missense mutation (rs121913529), 80.6% patients carrying a C/C genotype fell in the “moderate” subtype, whereas 56.8% patients with the variation C/T were in the “aggressive” subtype. According to the dbSNP database from the National Institutes of Health (Sherry, 2001), rs12193529 has an effect on KRAS protein variants G12D and GLY12ASP and is associated with the growth and metastasis of pancreatic tumor (Rachagani et al., 2011; Raphael et al., 2017). Furthermore, we confirmed that rs12193529 was indeed related to poor survival outcomes by performing a univariate Cox-PH analysis (HR = 2.51, *p* = 0.0011). With the multivariate Cox-PH test, we further observed that rs12193529 still had a significant impact on patient OS outcomes in addition to the MODEL-P subtypes (HR = 1.84, *p* value = 0.046).

**Figure 5:**
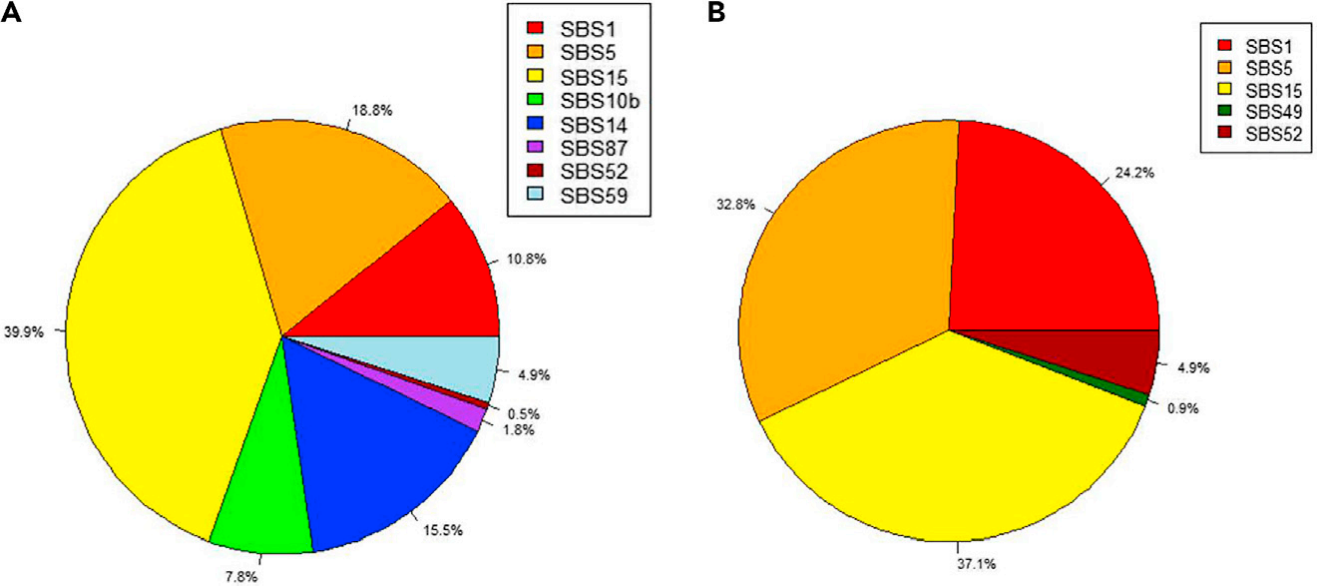
The percentage of each identified single-base substitution (SBS) signature in MODEL-P subtypes (A) The “moderate” subtype. (B) The “aggressive” subtype.

Second, potential etiologies associated with the identified subtypes were explored. More specifically, we applied SigProfileExtractor from COSMIC (Alexandrov et al., 2020) on the SNVs data of 140 samples. This tool decomposes the mutational profile of the input samples into known mutational signatures with validated etiologies. Using GRCh37 as the reference genome, we identified nine single-base substitution (SBS) signatures for the “moderate” subtype and five SBS signatures for the “aggressive” subtype (Figure 5). For more details of the results, Figure S5 shows the mutational profiles of the detected signatures for both subtypes and Table S2 summarizes the known etiologies of the found signatures.

In both subtypes, the SBS15 signature (Alexandrov et al., 2013) is the most frequent (39.9%, 37.1%, respectively). This mutational signature relates to a defective DNA mismatch repair and microsatellite instability, which appear to have a considerable impact on all patients with PDAC in our cohort. Of note, the clock-like signatures SBS1 (Nik-Zainal et al., 2012) and SBS5 (Alexandrov et al., 2013) are more frequent in the “aggressive” subtype (24.2%, 32.8%, respectively) than in the “moderate” subtype (10.8%, 18.8%, respectively), suggesting that aging could be a driver of the DNA repair defects in the “aggressive” subtype. More specifically, the SBS1 mutational signature is due to the failure of repairing/removing the mismatches of G:T in double-stranded DNA before DNA replication; the SBS5 signature is associated with the risk factor tobacco usage. Indeed, we observed marginal significant associations between age and the subtypes (*p* = 0.11) as well as between tobacco use and the subtypes (*p* = 0.17).

Uniquely to the “moderate” subtype, the polymerase epsilon-associated signatures 10b (Alexandrov et al., 2020) and SBS14 (Alexandrov et al., 2013) occur at frequencies (23.3% in total) comparable with those of the aging process-associated signatures (SBS1 and SBS5, 29.6% in total). The DNA polymerase epsilon is important for the synthesis of DNA and the repair of nucleotide and base excision (Bailey et al., 2016; Johnson et al., 2015; Lujan et al., 2016). Therefore, the dysfunction of the DNA polymerase epsilon might aggravate the deactivation of the DNA damage repair mechanism in the “moderate” subtype on top of the age-associated signatures. Moreover, we detected the SBS87 mutational signature (Li et al., 2020) in the “moderate” subtype, which is normally observed in the samples after using thiopurine chemotherapy to treat autoimmune diseases. This signature confirms the deficiency of the immune response in PDAC and infers that the lack of immune response for some “moderate” patients could be a consequence of previous drug usage.

## Discussion

High heterogeneity of PDAC has posed big challenges for prognosis prediction. In this study, we aimed at optimizing PDAC subtyping to help understand the prognosis-differentiating biological mechanisms. To achieve this, we developed a deep learning-based framework MODEL-P that defined two PDAC subtypes with distinct survival outcomes with log rank *p* = 1 × 10^−6^ based on three data modalities: mRNA, methylation, and microRNA expressions. Note that the number of subtypes was determined in a data-driven way by using the silhouette width and Calinski-Harabasz criterion. These two metrics suggested that, if the patients were split into two clusters, they had the most distinct survival outcomes between the clusters. To our knowledge, our study is the first attempt to integrate PDAC data with deep learning methods for patients' survival risk stratifications. The resulting subtypes (log rank *p* = 1 × 10^−6^) had much more distinct survival difference compared with the TNM stage (log rank *p* = 0.60) and tumor grade (log rank *p* = 0.44) on the TCGA training set. The AE model, which enabled the interaction of features with non-linear combination, outperformed the commonly used feature integration methods PCA and NMF (log rank *p* = 0.012 and 0.003, respectively). As for the contributions of the single omics data types to the multi-omics found clusters, mRNA was shown to be contributing more than microRNA and DNA methylation. Also, the clusters found by integrating all three omic data types have the most distinct survival outcomes (as compared with single or paired omics-based clusterings), indicating that all types of omics contributed to the prognosis differentiation from different aspects. In the future, we plan to include more types of data, and we are particularly interested in the whole slide images (WSIs). Studies (Cheerla and Gevaert, 2019; Peng et al., 2020) have demonstrated that WSI data alone, as well as together with genomic data, can achieve a remarkable performance in cancer prognosis prediction. However, most pancreatic cancer-specific studies using WSI data focused on diagnosis, i.e., pancreatic cancer detection and segmentation (Fu et al., 2021; Kriegsmann et al., 2021; Le et al., 2019). The predictive value of WSI for prognosis purpose has not been rigorously shown in pancreatic cancer. Once the prognostic value of WSI is validated in pancreatic cancer settings, we can evaluate the added predictive power compared with our benchmarked MODEL-P.

We also demonstrated that the MODEL-P subtypes were more predictive for patient prognosis, compared with the subtypes identified without the supervision of survival outcomes and by other approaches in previous studies. Previous PDAC subtyping studies made use of unsupervised approaches to cluster PDAC samples based on genomic profiles, mostly mRNA expression data, and showed that identified subtypes correlate to PDAC biological mechanisms. For example, Bailey et al. (2016) discovered four subtypes of PDAC with a *p* value of 0.030 based on mRNA-seq of 96 pancreatic cancer samples; Moffitt et al. (2015) defined two PDAC tumor-specific subtypes with a *p* value of 0.007 based on mRNA microarray data from 145 primary tumors; Collisson et al. (2011) had three subtypes with a *p* value of 0.039 based on mRNA microarray of 62 PDAC samples; Dijk et al. (2020) identified four subtypes with a *p* value of 0.002 based on the RNA-seq data of 90 PDAC samples; Yin et al. (2021) identified four subtypes with a *p* value of 9.2 × 10^−4^ using the DNA methylation data of 178 pancreatic cancer samples. Sinkala et al. (2020) defined two subtypes with a *p* value of 0.180 based on protein, mRNA, microRNA, and DNA methylation data of 45 high-purity pancreatic tumors. However, all of the subtypes defined by the unsupervised approaches had a much smaller survival difference than the prognosis-correlated subtypes defined by MODEL-P. More importantly, none of them has been validated for prognosis prediction on external test sets, which means the prognostic value of their subtypes for new patients is still unpredictable.

We applied AE to integrate multi-omics data in order to identify and define the subtypes from comprehensive perspectives. For prediction purpose, we chose to train and test on single omics data because single omics-based classifiers are more realistic and flexible applications for real-life decision-making process: instead of measuring the entire multi-omics, the clinicians can measure a certain type of omics (or panel) that is more feasible given the available resources and costs. Thus, we tested the two prognosis-correlated subtypes rigorously on external single-omics datasets. This was done by training the SVM prediction models based on the two identified subtypes and making subtype prediction on the independent five test sets. Patients in all test sets were successfully classified into two significantly distinct survival groups: the log rank *p* values for ICGC PACA-AU datasets were 0.030 for mRNA-seq, 0.031 for mRNA microarray, and 0.036 for methylation array dataset; the log rank *p* value for GEO: GSE62452 mRNA microarray was 0.007 and for GEO: GSE62498 microRNA was 0.029.

The pathway analysis was conducted by comparing the mRNA expression profiles of the two identified subtypes. Our pathway studies showed that the “aggressive” and “moderate” subtypes had different regulated extents of the DNA damage repair pathways and immune response pathways, respectively, which is in line with previous studies. Since PDAC is known for the deficiency of the immune response, and the immune cell interactions with the tumor cells remarkably affect tumor progression (Inman, 2014), it makes sense to observe that the immune response-related pathways are relatively more down-regulated in the “aggressive” subtype compared with the “moderate” subtype. Meanwhile, according to our mutational signature analysis, mutations related to age and tobacco use are present in both subtypes but are relatively more abundant in the “aggressive” subtype. This might explain the relatively more active DNA damage repair pathways in the “aggressive” subtype.

One KRAS missense mutation was found to be associated with OS as well as the MODEL-P subtypes. Moreover, the absence/presence of the mutation remained significant to predict OS in addition to the MODEL-P subtypes (in a multivariate Cox-PH regression). Moreover, we found that preoperative clinical risk factors did not add extra predictive value on top of identified prognosis-correlated subtypes. Not surprisingly, we did find that negative resection margin status (R0), in which no residual tumor is found, is associated with an improved survival outcome compared with R1 status. These results suggest that the subtypes identified by the three different types of omics data already cover the information contained in the clinical risk factors before the surgery. This is encouraging because it increases the clinical utility of our approach to support decision-making before surgery or other severe treatments, by profiling needle biopsy samples, for example. Furthermore, the mutational signature analysis showed that all patients with PDAC are closely associated with various types of DNA damage repair failures. Specifically, for patients in the “aggressive” subtype, the aging process-related repair defects and tobacco smoking play a major role. For the patients in the “moderate” subtype, the repair defects are related to clock-like and tobacco usage-related signatures as well as DNA polymerase epsilon mutations. Previous study (Haradhvala et al., 2018) has also demonstrated that these two types of signatures actually result in two distinct mechanisms of DNA repair defects in cancers, which might explain the distinction of prognosis for patients in different subtypes. Besides, this analysis also suggests that the treatments that inhibit the immune response might be the causation/have accelerated the oncogenesis of PDAC for some patients.

Taken together, our MODEL-P framework will facilitate PDAC subtype exploration and provide more effective support in personalized PDAC treatment to determine whether beneficial effects of the treatment or surgery outweighs the adverse events.

### Limitations of the study

To improve the robustness of the deep learning model, we need the addition of more samples that have better curation than the TCGA dataset, which contained patients who died of other reasons instead of PDAC (e.g., surgical complications), and the ICGC test sets contained patients who had already received chemotherapy and radiotherapy before surgery. In addition, since the training and test sets were from public databases, there may be a discrepancy of preprocessing carried out by the data generators, which may decrease the accuracy of prognosis prediction. For example, in the GEO datasets, the mRNA array dataset was provided with each probeset summarized by their mean expression values, whereas in the TCGA training set, each gene of mRNA-seq was summarized by the maximum values. Also, the microRNA data from the GEO database was derived from the NanoString technique, which was different from the TCGA dataset. Another limitation of this study is that the TCGA training set mainly contained patients in TNM stage II, whereas most of the pancreatic cancer tumors in clinics have already progressed into more advanced stages. We will enhance MODEL-P with ongoing multi-omics data collected from prospective studies in our institute to include more advanced tumor stages.

## STAR★Methods

### Key resources table

**Table:** 

REAGENT or RESOURCE	SOURCE	IDENTIFIER
**Deposited data**		

mRNA-seq of 146 PDAC primary tumor patients	The Cancer Genome Atlas	PAAD
microRNA-seq of 146 PDAC primary tumor patients	The Cancer Genome Atlas	PAAD
DNA methylation array of 146 PDAC primary tumor patients	The Cancer Genome Atlas	PAAD
mRNA-seq of 59 PDAC primary tumor patients	International Cancer Genome Consortium	PACA-AU
mRNA microarray of 64 PDAC primary tumor patients	International Cancer Genome Consortium	PACA-AU
DNA methylation array of 57 PDAC primary tumor patients	International Cancer Genome Consortium	PACA-AU
mRNA microarray of 65 PDAC primary tumor patients	Gene Expression Omnibus	GEO: GSE62452
microRNA array of 65 PDAC primary tumor patients	Gene Expression Omnibus	GEO: GSE62498

**Software and algorithms**		

TCGA-Assembler	Zhu et al., 2014	https://github.com/compgenome365/TCGA-Assembler-2
Survival	R Core Team	https://www.R-project.org
Survminer	R Core Team	https://www.R-project.org
biomaRt	Durinck et al., 2009	https://doi.org/10.18129/B9.bioc.biomaRt
Impute	Trevor et al., 2019	https://doi.org/10.18129/B9.bioc.impute
illuminaHumanv4.db	Dunning et al., 2015	https://doi.org/10.18129/B9.bioc.illuminaHumanv4.db
FDb.InfiniumMethylation.hg19	Tim Triche, 2015	https://doi.org/10.18129/B9.bioc.FDb.InfiniumMethylation.hg19
tensorflow 1.15.0	Abadi et al., 2016	https://www.tensorflow.org/
numpy 1.19.5	Harris et al., 2020	https://numpy.org/
scikit-learn 0.23.1	Pedregosa et al., 2011	https://scikit-learn.org/
pandas 1.1.2	McKinney, 2010	https://pandas.pydata.org/
MODEL-P code	This paper	https://github.com/ErasmusMC-Bioinformatics/MODEL-P

### Resource availability

#### Lead contact

Further information and requests for resources should be directed to and will be fulfilled by the lead contact, Yunlei Li (y.li.1@erasmusmc.nl)

#### Materials availability

This study did not generate new unique reagents.

### Experimental model and subject details

#### Datasets and cohorts

The TCGA multi-omics training set and five single omics external test sets were preprocessed prior to subtype identification and patient classification.

##### TCGA PAAD cohort

From TCGA PAAD cohort, we downloaded the mRNA-seq, microRNA and DNA methylation array data of 177 patients with R package TCGA-Assembler (Zhu et al., 2014). The mRNA-seq and microRNA were generated on Illumina HiSeq platform while DNA methylation data was derived from Illumina Infinium HumanMethylation450 BeadChip platform. According to TCGA, the mRNA-seq was processed by Expectation Maximization (RSEM) and normalized already (Li and Dewey, 2011). The microRNA-seq data was already normalized by reads per million (RPM). We selected patient samples with all three types of multi-omics data and clinical information available. Samples that were collected from the non-primary tumor tissues or other subtypes of pancreatic cancer were filtered out, with 146 PDAC primary tumor tissue samples remaining for our study. EtrezIDs of mRNA and CpG sites of DNA methylation were mapped to HUGO Gene Nomenclature Committee (HGNC) gene symbols. The CpG sites or EtrezIDs that could not be mapped to any gene symbols were removed, and the maximal measurement values were kept for those genes with multiple measurements. Genes from DNA methylation dataset with more than 20% of missing values and genes from mRNA and microRNAs with more than 20% of zero values among 146 samples were removed (Chaudhary et al., 2018; Zhu et al., 2017). We then filled in the remaining missing values for methylation genes using R package impute (Trevor et al., 2019). Log2(x+1) transformation was applied to mRNA and microRNA values to remove the domination of features with extremely large values. After preprocessing, the datasets contained 17188 mRNA features, 429 microRNA features, and 20980 DNA methylation features.

##### ICGC PACA-AU cohort

From ICGC PACA-AU cohort (2010), we downloaded three types of data, including mRNA sequencing of 59 samples, mRNA microarray of 64 samples and DNA methylation array of 57 samples. For each type of data, the samples were derived from primary tumor tissues of patients with PDAC.

For the ICGC-AU mRNA-seq dataset, the data has been normalized with Transcripts per Million (TPM) by the data generators. We then converted the Ensemble gene IDs to official HGNC gene symbols using R package biomaRt (Durinck et al., 2009). Ensemble gene IDs that could not be mapped to any gene symbols were filtered out and each gene was summarized with its highest value if multiple matched probe sets existed. Also, genes with more than 20% zero values were removed. Log2(x+1) transformation was applied on the data prior to use.

For the ICGC PACA-AU mRNA microarray dataset, the data has been normalized by Robust Multiarray Average (RMA) by the data generators. The Illumina probesets were mapped to official HGNC gene symbols using R package illuminaHumanv4.db (Dunning et al., 2015). Probesets that could not be mapped to any genes were filtered out and the highest value of each gene was kept if multiple probesets were present.

For the ICGC PACA-AU DNA methylation array dataset, the CpG islands were annotated with the transcription start sites (TSS) of genes within distance of 1500 base pairs with the official HGNC gene symbols using R package FDb.InfiniumMethylation.hg19, based on which the methylation beta values were averaged (Chaudhary et al., 2018; Tim Triche, 2015). Genes were summarized by their highest values of matched probesets. Those genes with more than 20% missing values were filtered out, and the remaining missing values were imputed using R package impute (Trevor et al., 2019).

After data preprocessing, the features overlapping with those in the TCGA training set were kept.

##### Datasets from NCBI GEO database

From NCBI GEO database, we obtained a cohort containing an mRNA microarray dataset and a microRNA dataset, under accession IDs GEO: GSE62452 and GEO: GSE62498 (Yang et al., 2016) respectively. In this cohort, there were 65 PDAC patient samples with primary tumor.

For the GEO: GSE62452 mRNA microarray dataset, derived from Affymetrix GeneChip platform, was already RMA-normalized and each gene was summarized by averaging the expression values of multiple corresponding probesets by the data generators. Before the data was further used, log2(x+1) transformation was applied.

For the GEO: GSE62498 microRNA dataset, the data was derived from Nanostring nCounter Platform. The microRNAs were already normalized per feature by the geometric mean. Log2(x+1) transformation was performed before further processing.

Following preprocessing, the features overlapping with those in the TCGA training set were kept for further process.

### Method details

#### Data integration by deep learning

An AE model (Kramer, 1991) was constructed on the TCGA training set to compress the original multiple types of omics features. Before integration, the L2 unit normalization was applied to scale all features of a certain type of omics in one sample into the range [0, 1] with the formula below:

$${\text{x}}_{\text{norm}}=\frac{\text{x}}{\sqrt{\sum_{\text{k}=1}^{\text{n}}|{\text{x}}_{\text{k}}{|}^{2}}}$$

Where x and ${\text{x}}_{\text{norm}}$ are the original and normalized feature values of a sample respectively; n is the number of features for a certain omics type in one sample.

The three types of normalized multi-omics data of 146 PDAC samples were concatenated and served as the input of the AE model. As an unsupervised neural network, AE consists of an encoding part to compress the features and a decoding part to reconstruct the encoded information. In this way, the model enables to capture important information and removes the noise in the original data source.

In this experiment, we constructed 3 hidden layers, including one bottleneck layer in the middle. The bottleneck layer was the result of feature extraction and was used in the subsequent steps. We employed activation function Tanh to transform the input values into [-1, 1]. During the training, the weight matrix was updated by stochastic gradient descent optimizer (Robbins and Monro, 1951). To measure the loss of transformation, we used Mean Squared Logarithm Error as the loss function. To prevent the overfitting of the Autoencoder (AE) model, we performed 4-fold cross validation (CV) with a random split on the trainin set to search for the optimal parameters. In each round of CV, we constructed a distinct AE model using three folds of the dataset as the training folds to identify the prognosis-correlated clusters. The omics features associated with prognosis-correlated clusters in the original space were selected to train the SVM models to classify the patients in the remaining hold-out fold into one of the identified clusters. The combination of AE parameters that predicted the most distinct clusters in the test folds of the CV was used to identify the PDAC subtypes based on all 146 patient samples in the training set. The parameters used in the final model included two hidden layers of 500 neurons, one bottleneck layer of 200 neurons, a batch size of 1, and epochs of 10 with a dropout rate of 0.5. Additionally, we added kernel regularization of 0.001 and activity regularization of 0.0001 to prevent overfitting of the AE. This model was built in python *TensorFlow1.15* (Abadi et al., 2016), *numpy1.19.5*(Harris et al., 2020) and *Pandas1.1.2* (McKinney, 2010).

#### Prognosis subtype identification

After 200 transformed features were extracted by AE from its bottleneck layer, a univariate Cox proportional hazards (Cox-PH) model was built to assess the correlations between the transformed features and patients’ overall survival (OS) outcomes. Fourteen transformed features were found to be significantly correlated (*p*-value < 0.05), based on which *K*-means clustering was performed to identify PDAC prognosis-correlated subtypes. We set the potential number of clusters from 2 to 5, and searched for the optimal number of clusters using two evaluation metrics: silhouette width, which estimated the distance between clusters (Rousseeuw, 1987), and Calinski-Harabasz criterion, which is the ratio of distances within clusters and between clusters (Calinski and Harabasz, 1974).

#### Prognosis subtype prediction

Single omics SVM classifiers were implemented to stratify new patients with PDAC in five external single omics test sets into diverse prognosis groups. Since classification in the transformed feature space would require all the multi-omics features in the TCGA training set to be measured for every new patient, it is practically more efficient to identify single omics subtype signatures in the original space to enable pathogenesis interpretation and prognosis prediction for new patients.

To begin with, each type of omics was normalized in the original feature space in two steps before further process. First, we applied Min-Max scaling per sample on each single omics set respectively to ensure that the same type of omics values derived from different platforms in the training and test sets were in the same range and comparable; then we performed robust scaling per feature to remove outliers and prepare data for prediction:

$${\text{x}}_{\text{norm}}=\frac{{\text{x}}_{\text{i}}-{\text{x}}_{\text{median}}}{{\text{quartile}}_{75}-{\text{quartile}}_{25}}$$

Where $x_{i}$ , $x_{median}$ and ${\text{x}}_{\text{norm}}$ are the original, median, and normalized values of a feature respectively. This was realized by RobustScaler function from python scikit-learn preprocessing (Pedregosa et al., 2011).

Subsequently the subtype signatures were selected by ANOVA F-test whereby features that were significantly associated (FDR < 0.05) with identified subtypes were ranked based on their ANOVA F-scores. The potential numbers of subtype signatures were set to be 5 to 200 features with a stepwise of 5, within which the number of signatures that gave the best average accuracy based on the 3-fold CV on the training set were used as the subtype signatures of each data type.

To predict patient prognosis in the external test sets, the subtype signatures present in each test set were first normalized in the same two-step manner in the training and the test set. ANOVA-F test was applied on this normalized training set to select test set-specific predictors. For each test set, a SVM model was built based on its specific predictors in the TCGA training set. The best combination of hyperparameters for SVM models were determined by grid search with 3-fold CV on the TCGA training set and then used for model construction on the entire training set. This section was implemented using python *scikit-learn* (Pedregosa et al., 2011).

### Quantification and statistical analysis

We evaluated the performance of MODEL-P for prognosis-correlated subtype identification in the TCGA training set and prediction in five external test sets by the log rank tests. The log-rank test assesses the significance of the survival difference between different groups, in our case the prognosis-correlated subtypes. Log rank p values were calculated from the Cox-PH model based on the actual OS and identified subtype labels. R package *survival* and *survminer* were used, also for plotting the Kaplan-Meier survival curves (Kaplan and Meier, 1958; Kassambara et al., 2017; Therneau and Grambsch, 2000).

## Data Availability

• This paper analyzes existing, publicly available data. These accession numbers for the datasets are listed in the key resources table. • The code generated during this study has been deposited at https://github.com/ErasmusMC-Bioinformatics/MODEL-P and is publicly available as of the date of publication. This has also been listed in the key resources table. • Any additional information required to reanalyze the data reported in this paper is available from the lead contact upon request.

## Supplemental information

Document S1. Figures S1–S5 and Tables S1 and S2

Data S1. mRNA, miRNA, and DNA methylation predictors of MODEL-P, related to STAR Methods
