# Supplementary material for: Robust deep learning model for prognostic stratification of pancreatic ductal adenocarcinoma patients
Source: iScience. 2021 Nov 10;24(12):103415. doi: 10.1016/j.isci.2021.103415 (PMC8637475; doi:10.1016/j.isci.2021.103415)
Supplement: Document S1. Figures S1–S5 and Tables S1 and S2 [file mmc1.pdf]

iScience, Volume *24*

Supplemental information

Robust deep learning model for prognostic stratification of pancreatic ductal adenocarcinoma patients

Jie Ju, Leonoor V. Wismans, Dana A.M. Mustafa, Marcel J.T. Reinders, Casper H.J. van Eijck, Andrew P. Stubbs, and Yunlei Li

Supplementary documents

Figure S1. The optimal number of clusters. This was the number of clusters that gave the largest scores by (A) silhouette width, and (B) Calinski-Harabasz methods. Related to Figure 2A and STAR Methods.

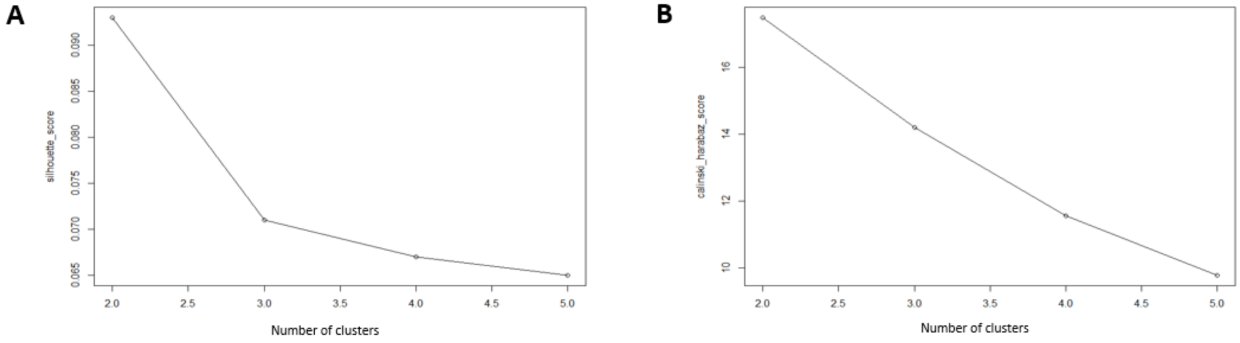

Figure S2. Survival differences between the prognosis-correlated subtypes identified using PCA and NMF for the multi-omics integration. Kaplan-Meier plots of the prognosis-correlated subtypes identified using A. PCA and B. NMF in the TCGA PAAD cohort. Related to Figure 2A.

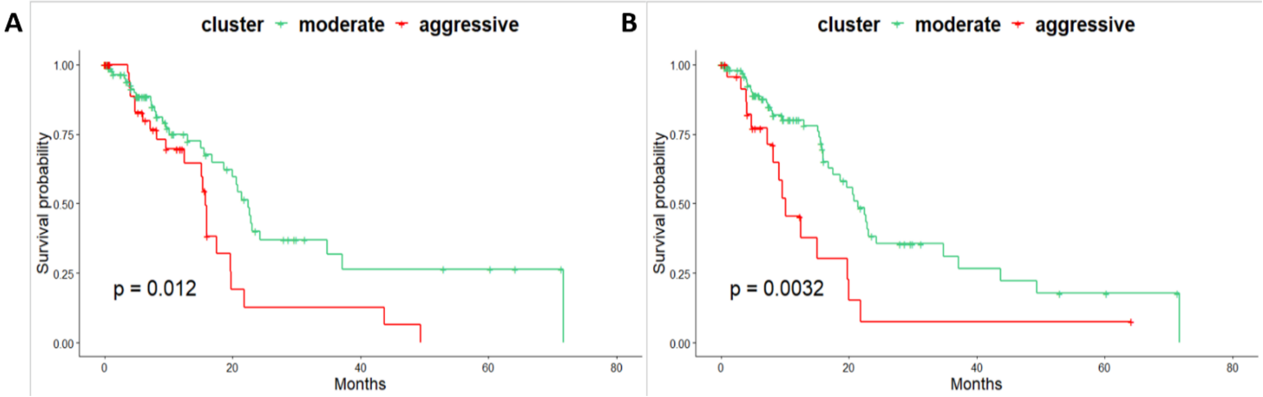

Figure S3. Impact of clinical risk factors on patient overall survival. The results of the univariate Cox-PH analysis (see Results) were given, which exhibited the impact of the clinical factors on patient actual OS individually. Related to Table 1.

Variable		N	Hazard ratio		p
tobacco_usage	CURRENT NON-SMOKER	101		Reference	
	CURRENT SMOKER	17		1.39 (0.65, 2.98)	0.391
age		146		1.02 (0.99, 1.04)	0.158
gender	FEMALE	68		Reference	
	MALE	78		1.14 (0.67, 1.97)	0.625
alcohol_usage	NO	50		Reference	
	YES	84		1.11 (0.61, 2.04)	0.730
diabetes_diagnosis	NO	88		Reference	
	YES	33		0.98 (0.50, 1.92)	0.958
chronic_pancreatitis	NO	105		Reference	
	YES	11		1.07 (0.45, 2.52)	0.878
residual_tumor	R0	83		Reference	
	R1	47		2.37 (1.31, 4.28)	0.004
	R2	5		0.54 (0.07, 3.95)	0.541
T	T1	4		Reference	
	T2	15		2.98 (0.35, 25.62)	0.319
	T3	123		2.84 (0.39, 20.74)	0.304
	T4	3		7.61 (0.46, 125.25)	0.155
N	T1	4		Reference	
	T2	15		2.98 (0.35, 25.62)	0.319
	T3	123		2.84 (0.39, 20.74)	0.304
	T4	3		7.61 (0.46, 125.25)	0.155
M	M0	69		Reference	
	M1	3		0.00 (0.00, Inf)	0.997
stage2	Stage I	12		Reference	
	Stage II	127		1.28 (0.46, 3.58)	0.638
	Stage III	3		3.39 (0.37, 31.27)	0.281
	Stage IV	3		0.00 (0.00, Inf)	0.997
grade	G1	21		Reference	
	G2	83		1.61 (0.61, 4.22)	0.338
	G3	41		2.25 (0.83, 6.11)	0.110
	G4	1		1.94 (0.22, 16.94)	0.548

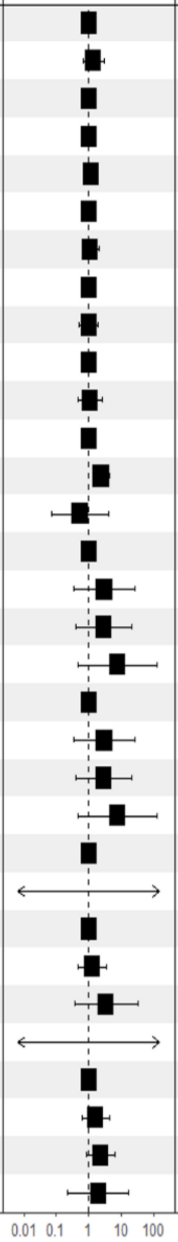

Figure S4. Added value of the clinical factors to identified subtypes. The results of the multivariate Cox-PH analysis (see Results) were given, which showed how the clinical factors affect the OS when prognosis-correlated survival subtypes are held constantly. Related to Table 1.

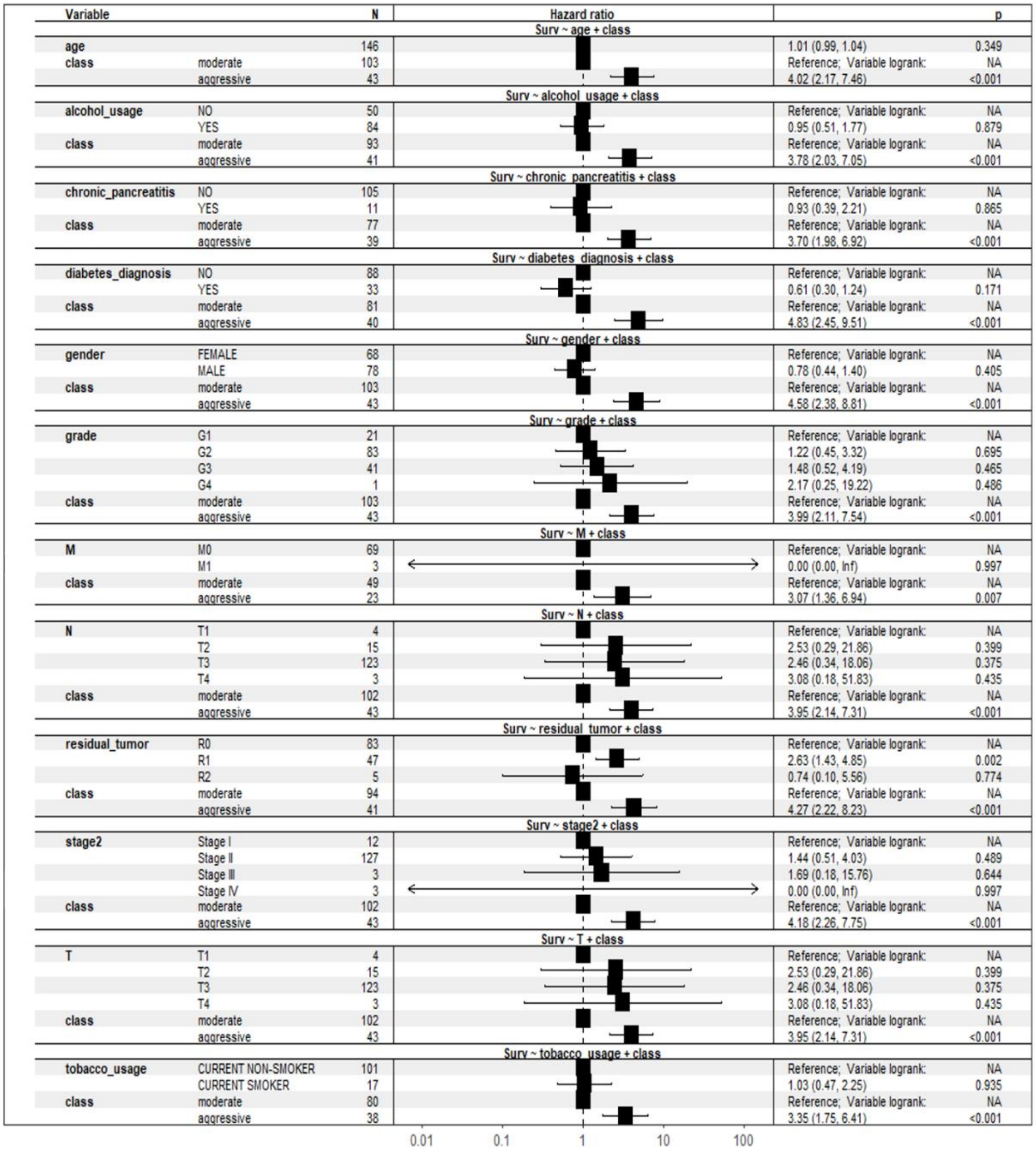

Figure S5. The mutational profiles of relevant single-base substitution (SBS) signatures in **A**. the “moderate” subtype, and **B**. the “aggressive” subtype. Related to Figure 5.

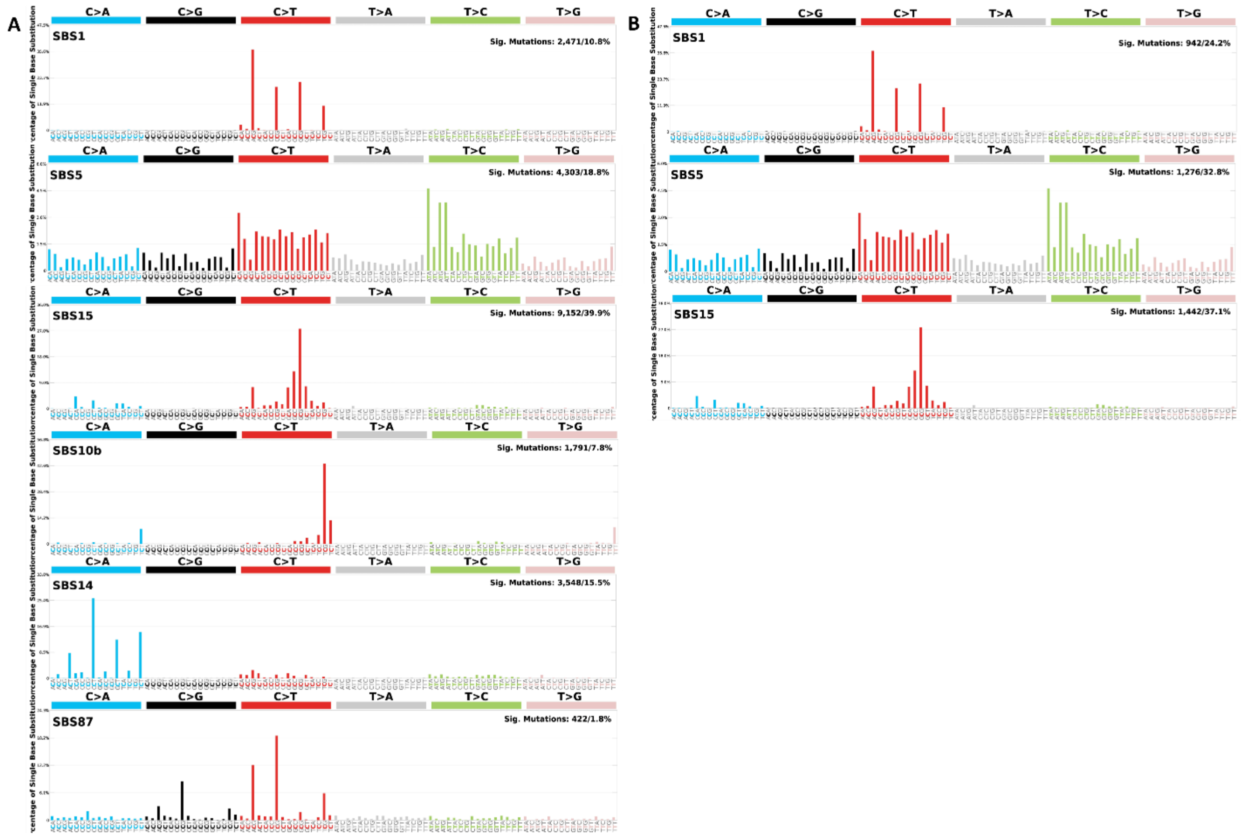

Table S1. Comparison between supervised prognosis-correlated approach and the unsupervised approach. Log-rank p-values of the two subtypes identified on the training set (TCGA PAAD) by these two approaches are shown. Based on identified subtypes, the survival difference of the predicted groups in the test sets are also given by log-rank p-values. Related to Figure 2.

**Datasets**	**Prognosis-correlated subtype identification and prediction**		**Unsupervised subtype identification and prediction**	
	**Number of predictors**	**Log-rank p-value**	**Number of predictors**	**Log-rank p-value**
**TCGA PAAD (n = 146)**	\	1e-6	\	0.005
**ICGC PACA-AU mRNA-seq (n = 59)**	107	0.030	83	0.500
**ICGC PACA-AU mRNA microarray (n = 64)**	99	0.031	66	0.050
**ICGC PACA-AU DNA methylation array (n = 57)**	81	0.036	17	0.400
**GEO GSE62452 mRNA microarray (n = 65)**	113	0.007	85	0.180
**GEO GSE62498 microRNA (n = 65)**	14	0.029	14	0.200

Table S2. The proposed etiologies of the single-base substitution (SBS) signatures according to COSMIC database. Related to Figure 5.

	**Proposed etiologies and comments**	**The percentage of the SBS in the "moderate" subtype**	**The percentage of the SBS in the "aggressive" subtypes**
**SBS1**	Clock-like mutational signature. This signature correlates with the individual's age, and might be a cell division/mitotic clock.<br>The mutational process is initiated by the G:T mismatches in double stranded DNA, which is caused by the spontaneous or enzymatic deamination of 5-methylcytosine to thymine causes. Then due to the failure to detect and remove these mismatches prior to DNA replication, the fixation of the T substitution for C occurs.	10.8%	24.2%
**SBS5**	Clock-like mutational signature. This signature correlates with the age and the tobacco smoking of the individual.	18.8%	32.8%
**SBS15**	Defective DNA mismatch repair.	39.9%	37.5%
**SBS10b**	Polymerase epsilon exonuclease domain mutations.	7.8%	0.0%
**SBS14**	Concurrent polymerase epsilon mutation and defective DNA mismatch repair.	15.5%	0.0%
**SBS87**	Thiopurine chemotherapy treatment, experimentally validated.	1.8%	0.0%
**SBS49**	Possible sequencing artefact.	0.0%	0.9%
**SBS52**	Possible sequencing artefact.	0.5%	4.9%
**SBS59**	Possible sequencing artefact.	4.9%	0.0%
